# Assessment of intestinal perfusion by a new balloon-tipped transpyloric probe

**DOI:** 10.1186/s13054-019-2326-8

**Published:** 2019-02-14

**Authors:** Samir G. Sakka

**Affiliations:** Department of Anesthesiology and Operative Intensive Care Medicine, University of Witten/ Herdecke, Medical Center Cologne Merheim, Ostmerheimerstr. 200, D-51109 Cologne, Germany

**Keywords:** Regional blood flow, Non-invasive assessment, Critically ill patients

Clinical tools for assessment of hepato-splanchnic perfusion and oxygenation are still very limited. Recently, a special balloon-tipped intestinal probe which is based on the principle of photoplethysmography (PPG) has been developed and was found to adequately detect an impairment of splanchnic perfusion in an experimental model of septic shock. Here, a critically ill patient is presented in whom as assessed by transpulmonary thermodilution systemic oxygen delivery increased following a fluid challenge while PPG indicated an improvement in splanchnic microcirculation. Further and larger clinical studies are required to investigate this relationship sufficiently.

Clinical assessment of hepato-splanchnic blood flow and oxygenation is still a major task. As has been previously shown, variables of regional blood flow and oxygen delivery are insufficient to estimate regional conditions [1]. While invasive procedures, e.g., liver vein catheterization, enable measurement of hepato-splanchnic blood flow, this approach does not adequately indicate local pathologies within the gastrointestinal tract [2]. Still, clinically applicable and reliable techniques are lacking which could help to rapidly identify and enable adequate steps to solve hepato-splanchnic hypoperfusion or hypoxia [3]. Recently, a special balloon-tipped intestinal probe which is based on the principle of photoplethysmography (PPG) has been found to be able to detect an impairment of the gut perfusion in an experimental model of septic shock [4, 5]. As the device has been clinically introduced, we here report first clinical data using this system in combination with other monitoring techniques in a critically ill patient.

A 54-year-old male with a subarachnoid hemorrhage (SAH) for aneurysm rupture was receiving mechanical ventilation, continuous monitoring of intracranial pressure, and advanced hemodynamic monitoring by transpulmonary thermodilution (PiCCO™, Pulsion, Getinge Group, Sweden). Due to SAH, mean arterial pressure was kept high (90–100 mmHg). For gastric reflux and disability to feed him enterally, a transpyloric trilumen probe had been placed by endoscopy. This particular probe (Ikorus™, APD, France) allows to assessing mucosal oxygenation as indicated by a PPG index. As the patient received advanced hemodynamic monitoring, we were able to follow changes during fluid administration (500 ml Jonosteril™, Fresenius Kabi, Germany) for clinical indication (Table 1).

**Table 1:** Different variables before and approx. 30 min after a fluid challenge

Variable	Prior to fluid challenge	After fluid challenge
Heart rate [1/min]	80	73
MAP [mmHg]	96	93
CI [l/min/m^2^]	3.3	3.9
PPV [%]	18	10
ITBVI [ml/m^2^]	1026	1080
NADR [μg/kg/min]	0.29	0.20
PPG	9.43	12.06

*MAD* mean arterial pressure, *CI* cardiac index, *PPV* pulse pressure variation, *ITBVI* intrathoracic blood volume index, *NADR* noradrenaline, *PPG* photoplethysmography

In this “responder” to fluid administration, findings may be interpreted as the optimization of cardiac preload (i.e., increase in intrathoracic blood volume and decrease in pulse pressure variation) and consecutively increase in cardiac output led to an optimized intestinal oxygenation as indicated by the PPG signal. In an animal study, the PPG signal has been found to reliably reflect the early perfusion alteration of the gut during sepsis [5]. Here, we support that optimized global flow may be associated with improved intestinal conditions. However, further and larger clinical studies are required to investigate this relationship sufficiently.
